# Validation of the Use of Automated and Manual Quantitative Analysis of Corneal Nerve Plexus Following Refractive Surgery

**DOI:** 10.3390/diagnostics10070493

**Published:** 2020-07-18

**Authors:** Jia Ying Chin, Lily Wei Yun Yang, Angel Jung Se Ji, Mario Nubile, Leonardo Mastropasqua, John Carson Allen, Jodhbir S. Mehta, Yu-Chi Liu

**Affiliations:** 1 Singapore Eye Research Institute, Singapore 168751, Singapore; jchin@tcd.ie (J.Y.C.); lily.yang@u.nus.edu (L.W.Y.Y.); angeljung32@gmail.com (A.J.S.J.); jodmehta@gmail.com (J.S.M.); 2 School of Medicine, Trinity College Dublin, D02 PN40 Dublin, Ireland; 3 Ophthalmic Clinic, University “G d’Annunzio” of Chieti-Pescara, Via dei Vestini, 66100 Chieti, Italy; nubilemario@gmail.com (M.N.); leonardo.mastropasqua@unich.it (L.M.); 4 Department of Biostatistics, Duke-NUS Medical School, Singapore 169857, Singapore; john.allen@duke-nus.edu.sg; 5 Department of Cornea and External Eye Disease, Singapore National Eye Centre, Singapore 168751, Singapore; 6 Ophthalmology and Visual Sciences Academic Clinical Program, Duke-NUS Medical School, Singapore 169857, Singapore

**Keywords:** corneal nerves, refractive surgery, automated quantification, manual quantification

## Abstract

Following refractive surgery, the cornea is denervated and re-innervated, hence a reproducible tool to objectively quantify this change is warranted. This study aimed to determine the repeatability and reproducibility of corneal nerve quantification between automated (ACCMetrics) and manual software (CCMetrics) following refractive surgery. A total of 1007 in vivo confocal microscopy images from 20 post-small incision lenticule extraction (SMILE) or post-laser-assisted in situ keratomileusis (LASIK) patients were evaluated by two independent observers using CCMetrics for corneal nerve fibre density (CNFD), corneal nerve branch density (CNBD), and corneal nerve fibre length (CNFL). Intra-observer and inter-observer reproducibility and repeatability, as well as agreement and correlation between the measurements obtained by ACCMetrics and CCMetrics, were assessed. We found that CNFL demonstrated the best intra- and inter-observer agreement followed by CNFD (intra-class correlation coefficient (ICC) = 0.799 and 0.740, respectively for CNFL; 0.757 and 0.728 for CNFD). CNBD demonstrated poorest intra- and inter-observer ICC. There was an underestimation in ACCMetrics measurements compared to CCMetrics measurements, although the differences were not significant. Our data suggested that both automated and manual methods can be used as reliable tools for the evaluation of corneal nerve status following refractive surgery. However, the measurements obtained with different methods are not interchangeable.

## 1. Introduction

The cornea has over 16,000 nerve terminations/mm^3^ and is the most densely innervated tissue in the human body [1]. Corneal nerves originate from the nasociliary branch of the ophthalmic division of the trigeminal nerve and divide into smaller branches that predominately innervate the anterior and middle of the stroma. Thereafter, they penetrate Bowman’s layer perpendicularly, then turn perpendicularly once again before branching into one or more subbasal nerves, forming the subbasal nerve plexus which runs between the Bowman’s layer and the basal corneal epithelium, parallel to the ocular surface. Corneal nerves consist of myelinated Aδ fibres of larger diameter and unmyelinated, beaded C fibres of smaller diameter, which provide important sensory functions and play an important role in maintaining the functional integrity of the ocular surface by releasing trophic mediators that promote corneal homeostasis [2,3].

Refractive errors are the leading cause of reversible visual impairment worldwide [4]. Laser refractive surgeries are the most common surgical procedures performed to correct refractive errors and achieve spectacle independence. Established as safe and effective procedures associated with excellent visual outcomes, improvements in quality of life, and high patient satisfaction [5], it is of no surprise that they are now one of the most commonly performed ophthalmic surgeries globally [6]. Laser in-situ keratomileusis (LASIK) and small incision lenticule extraction (SMILE) are two common laser refractive surgery techniques. In LASIK and SMILE, the flap or cap creation, stromal ablation/lenticule creation/extraction inevitably transect corneal nerves, leading to corneal denervation. This results in a decrease in corneal sensitivity and dry eye secondary to the neurotrophic cornea. Differences in surgical techniques between SMILE and LASIK are thought to have different repercussions on corneal nerve denervation and subsequent corneal nerve regeneration. The corneal nerve perforation sites, where the stromal nerve fibres perforate the Bowman’s layer and become the subbasal nerve plexus, are predominantly found in the mid-peripheral corneas [7]. Hence theoretically, in SMILE, with an incision of only 2.1–4 mm, as opposed to the circumferential flap (22 mm) in LASIK, the subbasal and stromal nerve fibres can be better preserved.

In-vivo confocal microscopy (IVCM) has been used extensively to study the cornea at a cellular level, and this includes the corneal subbasal nerve plexus. On IVCM evaluation, patients undergoing refractive surgery may present with a decrease in corneal nerve density, diameter and total length, as well as an increase in corneal tortuosity, branching and beading [8,9]. With the growing popularity of corneal refractive surgeries, much research has been devoted to minimising postoperative ocular surface disturbance via better preservation of corneal nerves through the development of new surgical technology and techniques [10]. Hence, there is a need for a reliable and reproducible quantitative tool to evaluate the extent of corneal denervation and subsequent nerve regeneration following refractive surgery. Assurance of the objective reproducibility of nerve metric evaluation would allow for reliable translation or correlation of corneal nerve analysis by IVCM images to subjective and objective clinical outcomes, such as dry eye assessments.

Currently, analytical software for corneal nerves range from being fully manual (CCMetrics; University of Manchester, Manchester, UK), semi-automated (NeuronJ; plug into ImageJ, NIH, Bethesda, Maryland, USA), to being fully automated (ACCMetrics; University of Manchester, Manchester, UK), all of which produce varying degrees of nerve quantification [11]. CCMetrics contains an interactive graphical interface which allows users to manually identify and trace nerve fibres, as a built-in algorithm accumulates and tabulates a number of nerve fibres and branches, as well as nerve fibre length and tortuosity [12]. Neuron J involves the manually-aided tracing of fluorescently labelled neurons using an algorithm which compares pixel intensity of neurons with neighbouring pixels, thus guiding manual tracing along an approximated length of neuron, in addition to tabulating quantities [13]. ACCMetrics uses a fully automated algorithm that enhances IVCM images, then distinguishes nerve fibres from the background using contrasting neighbouring pixels and quantifies them [14]. CCMetrics was previously used in several research papers to evaluate corneal diabetic neuropathy [15,16]. However, the laborious and time-consuming nature of the technique coupled with the need for trained expertise inspired the development of ACCMetrics, which utilises an algorithm to identify and quantify nerve fibres automatically and quickly. The convenient nature of ACCMetrics quickly led to its increasing popularity. Therefore, a comparison of different nerve analysis systems to determine the correlation and agreement of results between the software programmes would be beneficial in ensuring consistency in nerve quantification across different research work. This will allow for a fair investigation and comparison of nerve damage and regeneration following various surgical techniques, allowing optimisation of surgical techniques which can minimise nerve damage and resultant ocular surface disturbance in high-risk patients.

In the present study, we aimed to validate the use of automated and manual quantitative analysis of the corneal nerve plexus for patients who had undergone refractive surgery. We first evaluated the repeatability and reproducibility of manual quantitative analysis and then assessed the agreement and correlation between the automated and manual quantitative analysis.

## 2. Materials and Methods

The population of this study consisted of 20 patients (40 eyes) who were randomised to receive SMILE in one eye and LASIK in the other eye, performed in the Singapore National Eye Centre during the period between May 2012 and November 2016. This study was part of a registered randomised controlled trial (RCT; NCT01216475). Approval for the study was granted by the institutional review board of SingHealth, Singapore (reference number: 2011/109/A), and the study was conducted in accordance with the Declaration of Helsinki. LASIK and SMILE procedures were performed as previously described [17,18]. In brief, a superiorly hinged 120 μm thick flap was created using the Visumax femtosecond laser (Carl Zeiss, Jena, Germany) in the LASIK procedure, followed by excimer laser ablation (Wavelight Allegretto WAVE Eye-Q 400 Hz, Wavelight GmbH, Alcon, USA). For the SMILE procedure, the Visumax femtosecond laser with the following parameters was used—3.2 mm incision, 120 μm cap thickness, 7.5 mm cap diameter, 6.5 mm optical zone, and 145 nJ power with side-cut angles at 90 degrees. After careful dissection of the anterior and posterior planes of lenticule, the lenticule was extracted through the small incision.

The IVCM examination was performed at an average time of 4.3 ± 1.7 years after surgery (range 2.1–6.5 years). IVCM was conducted using a confocal scanning microscope, the Heidelberg HRT3 Rostock Cornea Module (Heidelberg Engineering GmbH, Heidelberg, Germany). Images generated were 2-dimensional, consisting of 384 × 384 pixels covering a field of 400 × 400 um. The cornea of the patient being examined was instilled with a drop of topical anaesthetic 0.4% benoxinate hydrochloride (Oxybuprocaine hydrochloride, Minims; Bausch and Lomb). The patient was then instructed to fixate on the flashing light of the instrument. The objective tip was advanced forward until gentle contact was established between the gel and the cornea. Both corneas of each patient were examined in five different areas: The central cornea was scanned first, and then the patient was asked to change their gaze to scan the superior, inferior, nasal and temporal part of the cornea (approximately 3 mm away from the corneal apex for each). The patients were instructed to fixate on a light source from a different direction with the contralateral eye to stabilise the scanning view.

For each scanned area, five best focused and most representative images from different depths of the subbasal nerves were selected. Each nerve (main trunk or branched nerve) was selected only once (i.e., the same nerve fibres were not repeatedly selected) to get a better representation of the subbasal nerve plexus. The 25 selected IVCM micrographs for each eye were evaluated using both manual and automated image analysis software (CCMetrics and ACCMetrics, respectively; University of Manchester, Manchester, UK). All the images were evaluated by two trained, independent, masked observers (JYC, LWYY) with CCMetrics for three nerve parameters: Corneal nerve fibre density (CNFD; number of main nerve fibres/mm^2^); corneal nerve fibre length (CNFL; total length of all nerve fibres in mm/mm^2^); and corneal nerve branch density (CNBD; number of branch points on the main fibres/mm^2^), and inter-observer reproducibility was evaluated. In CCMetrics measurements, all visible nerves were traced with a manual drawing module, by manually marking the nerve fibres with red lines, nerve branches with blue lines, and by putting green dots to the branching points, defined as the points intersecting the fibres and branches. Two weeks after the completion of the first evaluation, both observers repeated the analysis for all the images, and intra-observer repeatability was assessed [19]. All the images were also analysed with ACCMetrics, and the agreement and correlation between the ACCMetrics and CCMetrics measurements was evaluated.

The data were analysed using STATA (STATACorp, TX, USA) and NCSS (LLC, MI, USA), and was presented as mean ± standard deviation. Bland-Altman plots were employed to determine intra- and inter-observer agreements between the measurements. The repeatability and reproducibility values were calculated in terms of mean bias and 95% limits of agreement (LoA). The intraclass correlation coefficient (ICC) was determined as an index of repeatability and reproducibility between measurements. Paired *t*-tests were used to assess the differences between intra-observer and inter-observer measurements. A Passing-Bablok regression was used to evaluate the relationship between the ACCMetrics and CCMetrics measurements. *p* < 0.05 was considered statistically significant.

## 3. Results

### 3.1. Patient Characteristics

**Figure 1:**
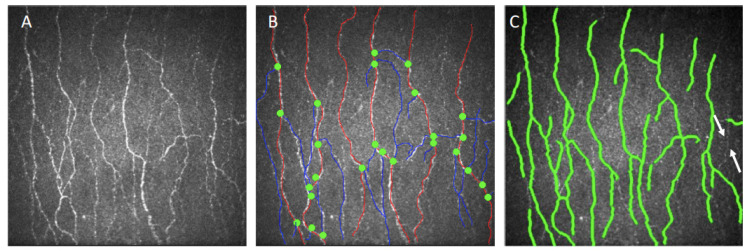
Illustrations representative of (**A**) raw IVCM images, (**B**) images marked manually with CCMetrics and (**C**) images marked automatically with ACCMetrics. In the image analysed by CCMetrics, nerve fibres are marked by red lines, nerve branches are marked by blue lines, and branch points are marked by green dots. In the image analysed by ACCMetrics, a low contrast nerve fibre is missed out (white arrows).

The mean age at the time of surgery was 25.0 ± 4.8 years (female: male = 14:6). The mean corrected spherical equivalent was–4.65 ± 1.26 D and−4.78 ± 1.45 D for the SMILE and LASIK eyes, respectively. A total of 1007 images from these 20 patients were analysed with both CCMetrics and ACCMetrics (Figure 1). Table 1 summarises the data for CNFD, CNFL and CNBD.

### 3.2. Intra-Observer and Inter-Observer Agreement of CCMetrics Measurements

The ICC for intra-observer and inter-observer measurements using CCMetrics are presented in Table 2. CNFL demonstrated the best intra-observer agreement among the three parameters with an average ICC value of 0.799 between the two observers, followed closely by CNFD, which demonstrated good intra-observer agreement with an average ICC value of 0.757. CNBD had the worst intra-observer agreement with an average ICC value of 0.653.

In a similar pattern, CNFL showed the best inter-observer agreement with an average ICC value of 0.740 between the two measurements. This was followed by CNFD, which showed very good inter-observer agreement with an average ICC value of 0.728. CNBD showed the worst inter-observer agreement with an average ICC value of 0.591. The ICC values of inter-observer measurements for all three parameters were lower than that of intra-observer measurements.

### 3.3. Intra-Observer Reproducibility and Inter-Observer Reproducibility of CCMetrics Measurements

**Figure 2:**
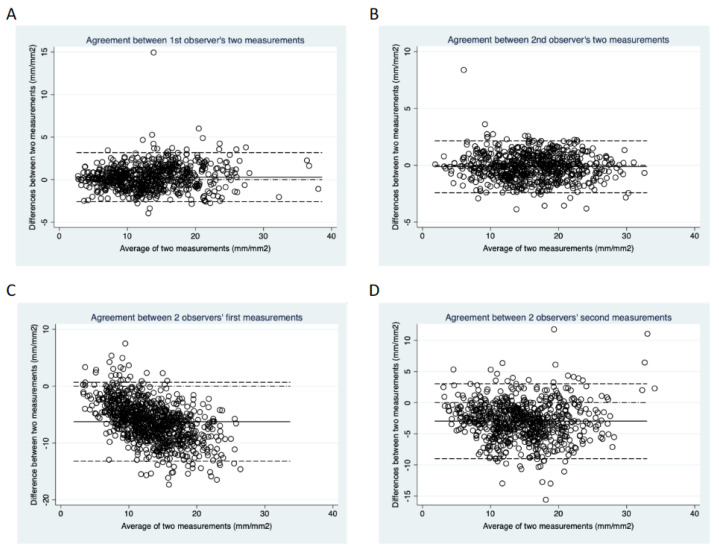
Bland Altman plots of repeated measurements of CNFL by (**A**) observer 1 and (**B**) observer 2 representing intra-observer repeatability. Bland Altman plots of CNFL by different observers for each measurement; (**C**) first measurement and (**D**) second measurement represents inter-observer reproducibility.

The intra-observer mean biases and LoA for observer 1 and 2 of the different parameters are presented in Table 3 and Figure 2A,B. All three parameters, CNFL, CNFD and CNBD showed no statistically significant intra-observer difference (all *p* > 0.05).

The inter-observer mean biases and LoA of the different parameters are presented in Figure 2C,D and Table 4. All three parameters, CNFL, CNFD, and CNBD, showed no statistically significant inter-observer difference. The mean bias of CNFL improved from−6.692 mm/mm^2^ in the first measurement to−2.513 mm/mm^2^ in the second measurement. Similarly, the mean bias of CNFD improved from−7.262 fibres/mm^2^ in the first measurement to−4.471 fibres/mm^2^ in the second measurement. In addition, the Bland Altman plot of the agreement between the two observers’ second measurements depicts a more symmetrical pattern about the line of mean difference compared to that of the two observers’ first measurement (Figure 2D), indicating that the second CNFL measurement demonstrated better inter-observer agreement than the first measurement. The dots in the Bland Altman plots of the agreement between observer 1′s two measurements and observer 2′s two measurements are closer to the zero line, compared to that of the two observers’ first measurements and the two observers’ second measurements, suggesting that inter-observer agreement is worse than intra-observer agreement for CNFL values.

### 3.4. Agreement of Parameters Measured by CCMetrics and ACCMetrics

The mean values of CNFL, CNFD and CNBD obtained using ACCMetrics were lower than those measured by CCMetrics (Table 1). The mean CNFL quantified by ACCMetrics was lower than the mean CNFL quantified by observer 1 and observer 2 on their second grading (more experienced grading), with mean differences of 2.5 and 5.0 mm/mm^2^, respectively. Likewise, the mean CNFD quantified by ACCMetrics was lower than the mean CNFD quantified by observer 1 and observer 2 on their second grading, with mean differences of 2.4 and 4.0 fibres/mm^2^. Similarly, the mean CNBD quantified by ACCMetrics was lower than the mean CNBD quantified by observer 1 and observer 2 on their manual grading, with mean differences of 8.2 and 7.2 branch points on the main fibres/mm^2^.

**Figure 3:**
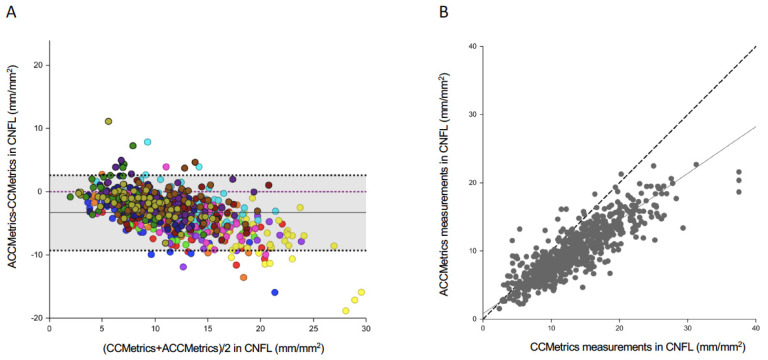
Plots depicting the (**A**) agreement and (**B**) Passing–Bablok correlation between CNFL measured by ACCMetrics and CCMetrics. In (**A**), the solid line represents the mean difference, and the black dashed lines represent the 95% limits of agreement. The dots in the same colour represent the values of 25 images from the same patient. In (**B**), the solid line indicates the regression line, while the dotted line indicates the equivalence line.

Bland-Altman plots of agreement and Passing-Bablok correlation between CNFL measured by ACCMetrics and CCMetrics are shown in Figure 3. The CNFL measurements obtained by ACCMetrics and CCMetrics demonstrated good agreement and significantly strong correlation, with the ICC of 0.789 (*p* < 0.01) and Passing-Bablok regression correlation coefficient of 0.821 (*p* < 0.01). ACCMetrics underestimated CNFL values by 3.752 mm/mm^2^ on average (mean bias; LOA−2.388 to 9.892, *p* = 0.367). This underestimation in ACCMetrics appeared to be more apparent (Figure 3A) with an increase in CNFL. The CNFD measurements also have a good agreement between ACCMetrics and CCMetrics software with an ICC of 0.811, while the agreement in CNBD was lower with an ICC of 0.642 (Table 2). Similarly, ACCMetrics underestimated CNFD and CNBD values compared to CCMetrics, with the mean bias of 4.65 fibres/mm^2^ (LOA−2.960 to 11.810, *p* = 0.241) and 7.70 branch points on the main fibres/mm^2^ (LOA−0.533 to 15.933, *p* = 0.104), respectively.

## 4. Discussion

In the present study, we demonstrated that both automated and manual nerve analytic software were reproducible and had good agreement in the evaluation of corneal nerves status following refractive surgery, particularly in the assessment of CNFL and CNFD. However, we found that automated ACCMetrics attained lower values compared to manual CCMetrics, especially when the fibre density or fibre length was higher. CNBD, compared to other parameters, had lower but still acceptable intra-observer and inter-observer reproducibility.

Existing nerve analysis software can be broadly categorised into manual, semi-automatic and fully automatic, each with varying disadvantages and advantages [11]. The three most commonly utilised nerve analysis software programmes in each category are CCMetrics for manual quantification, NeuronJ for semi-automated quantification and ACCMetrics for fully automated quantification. ACCMetrics utilises a fully automated algorithm which identifies and quantifies nerve fibres in eight different parameters, compared to the four parameters quantified by CCMetrics (CNFD, CNFL, CNBD and tortuosity coefficient). The four additional parameters, nerve fibre area, nerve fibre width, nerve fibre orientation histogram and nerve fibre width histogram, are advantageous in that they provide additional dimensions and allow for more detailed comparisons in nerve analysis. Automated quantification consists of two steps. Firstly, images are enhanced, and nerve fibres are detected by a dual model feature descriptor combined with a neural network classifier which distinguishes nerve fibres from background noise and underlying connective tissue [20]. Next, the software quantifies the morphometric nerve parameters. Each image takes an average of approximately 15 s for the complete analysis, much shorter than the 10–20 min taken for manually analysis depending on the complexity of the image [21]. The automated software is, thus, efficient in that it saves the analyst time, removes the need for an expert and exhibits objectivity in nerve identification, especially if nerve fibres are of good calibre and demonstrate good contrast from the background [22]. This would also eliminate the need to account for inter-observer and intra-observer variability and reproducibility in ascertaining the validity and reliability of the results.

However, IVCM corneal nerve images contain a wide range of nerve fibres of differing contrast from the background. Thicker nerve fibres tend to show stronger contrast from the background and are more easily identified by the automated algorithm. On the other hand, nerves that are not in focus, as well as thinner fibres that tend to appear fainter, may be missed out due to their poor contrast in especially in images with noisy backgrounds [22]. These false-negative errors may result in an underestimation of the nerve parameters. In contrast, manual software CCMetrics utilises an algorithm to tabulate nerve statistics following manual identification and tracing of nerve fibres on an interactive graphical interface of the nerve images. Nerve identification and tracing depends on the user’s trained discretion rather than automated algorithms. These differences in software explain the underestimation of nerve parameters by ACCMetrics seen in our study, in which we observed a greater percentage of underestimation error as CNFL values increased. ACCMetrics may also be associated with several overestimation scenarios occasionally, resulting from mistaken recognition of some non-nerve related structures, such as light reflections or dendritic cells that display sufficient contrast to fulfil the criteria of the algorithm, although these occurred less frequently in our experience.

However, manual marking is time-consuming and labour-intensive with an analysis time of approximately 20 min per image. Given its laborious nature, manual tracing is subject to user fatigue, increasing the possibility of unreliable tracing, and lowering productivity due to the limitation in the number of images analysed per day [23]. In addition, it is highly user-dependent and requires the expertise of the observer in both identifying and tracing the tortuous nerve contours. Thus, the subjective nature of nerve identification is more obvious in manual quantification, resulting in inconsistencies and a need for both inter-observer and intra-observer analysis [23]. This is illustrated by the worse inter-observer agreement compared to the intra-observer agreement. In our study, we also showed that the inter-observer agreement between the second measurements of all the parameters measured by CCMetrics was better than that between the first measurements. This is not surprising given that manual nerve tracing accuracy improves with user experience, as repetition and exposure allows for familiarity and refinement of nerve identification and tracing skills.

Several previous published studies have compared manual and fully automated methods of nerve quantification, but all of them have been limited to patients with diabetes mellitus (DM). The pathogenesis of DM corneal neuropathy is different from that of refractive surgery. DM causes a slow degenerative neuropathy [24], while corneal denervation following refractive surgery involves acute nerve denervation. Furthermore, DM patients show minimal nerve regeneration if any, even after systemic stabilisation. In contrast, post-refractive surgery patients demonstrate nerve regeneration, which may take several years [25]; hence, it is imperative that there exists a reliable analytic tool to quantify these changes. Since there is an inherent underlying difference in the mechanism of nerve damage and repair between refractive surgery and DM corneal neuropathy, it is important to study the validity between the automated and manual software to ensure consistency in nerve quantification in refractive surgery.

Dehghani et al. reported that automated methods produced underestimated CNFL results compared to manual methods in the patients with diabetic corneal neuropathy, but the results obtained from both methods were in good agreement and correlation, with an ICC value of 0.86 and Pearson correlation coefficient of 0.87 [22]. In our study, we showed good agreement in CNFL measurements obtained by CCMetrics and ACCMetrics, with an ICC of 0.789, which was lower than that reported by Dehghani et al. A possible reason for this might be the difference in the mechanism of repair of corneal subbasal nerves, which primarily results from the nerve regeneration capability following refractive surgery that does not occur in progressive DM. Thin and short regenerating nerves following refractive surgery have a higher tendency to be missed by ACCMetrics in our experience. In another study on DM corneal neuropathy, Ostrovsky et al. showed good correlation and agreement for CNFL between manual and automated software, with a Pearson correlation coefficient of 0.82 [21]. However, the authors pointed out that there was an absolute measurement bias, corresponding to a 21% underestimation of absolute CNFL values using ACCMetrics. In our paper, ACCMetrics had an average underestimation of 3.582 mm/mm^2^ in CNFL, corresponding to a 32% underestimation of absolute CNFL values. The higher underestimation in the present study might have arisen from the fact that our study population had higher CNFL (11.3 ± 5.8 mm/mm^2^; Table 1) than the DM population (9.58 ± 2.19 mm/mm^2^) [21], and the effect of underestimation is greater with the increase in CNFL values (Figure 3A).

CNFD measurements demonstrated good agreement between ACCMetrics and CCMetrics with an ICC value of 0.811. This supports the premise that ACCMetrics is capable of identifying well contrasted, thicker nerves, of which long main nerve fibres typically possess at least one characteristic along a significant portion of its length, and are thus, correctly identified as main nerves. CNFD measurements also demonstrated good intra-observer and inter-observer agreement using CCMetrics, with average ICC values of 0.757 and 0.728, respectively. There was little difference between manually obtained CNFD values as main nerves are typically long and easily identifiable manually. Small differences between observers arose mainly in images with many conjoined long nerves, in which there was a difference in opinion regarding whether the nerves were main nerves or long branching nerves.

Among the three parameters, CNBD showed the worst agreement between ACCMetrics and CCMetrics, as well as the worst intra-observer and inter-observer agreement in CCMetrics measurements with ICC values of 0.642, 0.653, and 0.591, respectively. This is consistent with a previous DM corneal neuropathy study, in which the authors demonstrated that intra-observer and inter-observer repeatability and consistency were better for CNFD and CNFL, compared to CNBD (inter-observer ICC 0.54; intra-observer 0.61) [26]. We postulate that the poor contrast of nerves from background contributed to the discrepancy in identifying branch points. ACCMetrics is sometimes unable to identify the full length of main nerves where they become thinner or fainter, causing it to miss branch points extending from that portion of the main nerve. In addition, branches which demonstrated poor contrast either in their full length or near their branch points were also not identified by ACCMetrics (Figure 1C). The poor agreement between observers and software can also be attributed to the variability regarding disputable nerve structures in which the distinction between clearly defined nerve parameters was unclear. Common problems involved two crossing fibres, which could be interpreted as a single branching fibre by one user and as two fibres without branches by the other user. These reasons could explain the propensity for errors in the CNBD measurements.

The inherent limitation of current IVCM scans is that it provides a small region of interest (<500 μm^2^) and is not easy to standardise scanning location and depth each time. The distribution of corneal nerve plexus also varies in the central and peripheral cornea [27]. Hence, it is important to standardise the scanning and analysing protocol when utilising IVCM for research. Automated mapping or montaging techniques for images [28], as well as wide-field or large-area scanning of corneal subbasal nerve plexus [29], may be helpful in the future.

## 5. Conclusions

In conclusion, we have demonstrated that both automated and manual nerve analytic tools had good repeatability and reproducibility in the nerve metrics measurements for patients following refractive surgery. In particular, CNFL and CNFD demonstrated good inter-observer and intra-observer agreement for CCMetrics, as well as between CCMetrics and ACCMetrics compared to CNBD. The ability of automated quantification to overcome the time-consuming, laborious, subjective biases of manual quantification, while maintaining reproducible results gives it an advantage over manual quantification of corneal nerves. However, as automated quantification underestimates nerve parameters compared to manual quantification, particularly when nerve fibres demonstrate poor contrast or are of small calibre, measurements obtained with different quantification methods are not interchangeable. The validation of quantitative methods in our study will further ensure accurate comparisons within or across research in nerve analysis demonstrating denervation and re-innervation.

## Figures and Tables

**Table 1:** Mean ± SD of parameters measured by ACCMetrics and CCMetrics.

	ACCMetrics	CCMetrics, Observer 1, First Measurement	CCMetrics Observer 1, Second Measurement	CCMetrics Observer 2, First Measurement	CCMetrics Observer 2, Second Measurement
CNFL	11.3 ± 5.8	13.3 ± 7.1	13.8 ± 6.2	19.9 ± 10.1	16.3 ± 9.8
CNFD	13.9 ± 7.7	15.2 ± 8.8	16.3 ± 8.5	20.8 ± 12.1	17.9 ±11.7
CNBD	17.0 ± 9.3	29.0 ± 17.6	25.2 ± 13.4	28.8 ± 17.3	24.2 ± 15.7

CNFL (corneal nerve fibre length) is measured in total length of fibre (mm/mm^2^). CNFD (corneal nerve fibre density) is measured in number of fibres/mm^2^. CNBD (corneal nerve branch density) is measured in number of branch points on the main fibres/mm^2^.

**Table 2:** The intraclass correlation coefficient (ICC) for measurements of CCMetrics and ACCMetrics.

ICC	CCMetrics								ACCMetrics	
	Intra-observer				Inter-observer				Comparison with CCMetrics *	
	Observer 1	*p* value	Observer 2	*p* value	First Measurement	*p* value	Second Measurement	*p* value		*p* value
CNFL	0.784 (0.444–0.912)	<0.01	0.813 (0.528–0.882)	<0.01	0.706 (0.242–0.859)	0.01	0.774 (0.255–0.802)	<0.01	0.789 (0.461–0.882)	<0.001
CNFD	0.788 (0.502–0.848)	<0.01	0.726 (0.647–0.913)	<0.01	0.647 (0.218–0.837)	0.01	0.808 (0.655–0.912)	<0.01	0.811 (0.754–0.903)	<0.001
CNBD	0.663 (0.482–0.755)	<0.01	0.642 (0.356–0.708)	<0.01	0.552 (0.203–0.706)	0.018	0.629 (0.275–0.806)	0.012	0.642 (0.422–0.757)	<0.001

* Comparisons between ACCMetrics and mean of second measurements from two observers. In brackets are the 95% confidence intervals. CNFL is measured in total length of fibre (mm/mm^2^). CNFD is measured in number of fibres/mm^2^. CNBD is measured in number of branch points on the main fibres/mm^2^.

**Table 3:** Intra-observer mean biases and limits of agreement (LoA) for parameters in manual marking (CCMetrics).

Nerve Parameters	Mean Bias	LoA	*p* Value *
Observer 1			
CNFL	0.574	−2.294 to 3.442	0.588
CNFD	1.082	−3.334 to 5.498	0.339
CNBD	3.803	−5.785 to 13.391	0.204
Observer 2			
CNFL	−0.118	−2.639 to 2.403	0.732
CNFD	1.695	−3.596 to 6.986	0.258
CNBD	3.792	−5.142 to 12.726	0.204

* The comparison of the two measurements of two time points from the same observer. CNFL is measured in total length of fibre (mm/mm^2^). CNFD is measured in number of fibres/mm^2^. CNBD is measured in number of branch points on the main fibres/mm^2^.

**Table 4:** Inter-observer mean biases and limits of agreement (LoA) for parameters in manual marking (CCMetrics).

Parameters	Mean Bias	LoA	*p* Value *
First Measurement			
CNFL	−6.692	−13.672 to 0.288	0.117
CNFD	−7.262	−16.626 to 2.102	0.143
CNBD	−6.807	−16.435 to 2.821	0.106
Second Measurement			
CNFL	−2.513	−8.888 to 3.862	0.350
CNFD	−4.471	−10.824 to 1.882	0.238
CNBD	−6.756	−13.618 to 0.106	0.212

* The comparison of the two measurements from two observers. CNFL is measured in total length of fibre (mm/mm^2^). CNFD is measured in number of fibres/mm^2^. CNBD is measured in number of branch points on the main fibres/mm^2^.

## References

[1] Guthoff R.F., Wienss H., Hahnel C., Wree A. (2005). Epithelial innervation of human cornea: A three-dimensional study using confocal laser scanning fluorescence microscopy. Cornea.

[2] Marfurt C.F., Cox J., Deek S., Dvorscak L. (2010). Anatomy of the human corneal innervation. Exp. Eye Res..

[3] Al-Aqaba M.A., Dhillon V.K., Mohammed I., Said D.G., Dua H.S. (2019). Corneal nerves in health and disease. Prog Retin Eye Res..

[4] Lou L., Yao C., Jin Y., Perez V., Ye J. (2016). Global Patterns in Health Burden of Uncorrected Refractive Error. Invest. Ophthalmol Vis. Sci..

[5] Solomon K.D., Fernandez de Castro L.E., Sandoval H.P., Biber J.M., Groat B., Neff K.D., Ying M.S., French J.W., Donnenfeld E.D., Lindstrom R.L. (2009). LASIK world literature review: Quality of life and patient satisfaction. Ophthalmology.

[6] Kim T.I., Alio Del Barrio J.L., Wilkins M., Cochener B., Ang M. (2019). Refractive surgery. Lancet.

[7] Al-Aqaba M.A., Fares U., Suleman H., Lowe J., Dua H.S. (2010). Architecture and distribution of human corneal nerves. Br. J. Ophthalmol..

[8] Mohamed-Noriega K., Riau A.K., Lwin N.C., Chaurasia S.S., Tan D.T., Mehta J.S. (2014). Early corneal nerve damage and recovery following small incision lenticule extraction (SMILE) and laser in situ keratomileusis (LASIK). Invest. Ophthalmol Vis. Sci..

[9] Patel D.V., McGhee C.N. (2009). In vivo confocal microscopy of human corneal nerves in health, in ocular and systemic disease, and following corneal surgery: A review. Br. J. Ophthalmol..

[10] Mastropasqua L., Nubile M., Sekundo W. (2015). Corneal nerve and keratocyte response to ReLEx surgery. Small Incision Lenticule Extraction (SMILE): Principles, Techniques, Complication management and Future concepts.

[11] Liu Y.C., Lin T.Y., Mehta J.S. (2020). Analysis of Corneal Nerve Plexus in Corneal Confocal Microscopy Images. Neural Regen. Res..

[12] Jiang T., Navab N., Pluim J.P., Viergever M.A. (2010). Medical Image Computing and Computer-Assisted Intervention—MICCAI 2010: 13th International Conference, Beijing, China, 20–24 September 2010.

[13] Popko J., Fernandes A., Brites D., Lanier L.M. (2009). Automated analysis of NeuronJ tracing data. Cytom. A.

[14] University of Manchester. ACCMetrics user instructions. http://research.bmh.manchester.ac.uk/ena/ACCMetricsuserinstructions/.

[15] Ferdousi M., Romanchuk K., Mah J.K., Virtanen H., Millar C., Malik R.A., Pacaud D. (2019). Early corneal nerve fibre damage and increased Langerhans cell density in children with type 1 diabetes mellitus. Sci. Rep..

[16] Kalteniece A., Ferdousi M., Azmi S., Mubita W.M., Marshall A., Lauria G., Faber C.G., Soran H., Malik R.A. (2020). Corneal confocal microscopy detects small nerve fibre damage in patients with painful diabetic neuropathy. Sci. Rep..

[17] Liu Y.C., Rosman M., Mehta J.S. (2017). Enhancement after Small-Incision Lenticule Extraction: Incidence, Risk Factors, and Outcomes. Ophthalmology.

[18] Ang M., Farook M., Htoon H.M., Mehta J.S. (2019). Randomized Clinical Trial Comparing Femtosecond LASIK and Small-Incision Lenticule Extraction. Ophthalmology.

[19] Liu Y.C., Konstantopoulos A., Riau A.K., Bhayani R., Lwin N.C., Teo E.P., Yam G.H., Mehta J.S. (2015). Repeatability and Reproducibility of Corneal Biometric Measurements Using the Visante Omni and a Rabbit Experimental Model of Post-Surgical Corneal Ectasia. Transl. Vis. Sci. Technol..

[20] Chen X., Graham J., Dabbah M.A., Petropoulos I.N., Ponirakis G., Asghar O., Alam U., Marshall A., Fadavi H., Ferdousi M. (2015). Small nerve fiber quantification in the diagnosis of diabetic sensorimotor polyneuropathy: Comparing corneal confocal microscopy with intraepidermal nerve fiber density. Diabetes Care.

[21] Ostrovski I., Lovblom L.E., Farooqi M.A., Scarr D., Boulet G., Hertz P., Wu T., Halpern E.M., Ngo M., Ng E. (2015). Reproducibility of In Vivo Corneal Confocal Microscopy Using an Automated Analysis Program for Detection of Diabetic Sensorimotor Polyneuropathy. PLoS ONE.

[22] Dehghani C., Pritchard N., Edwards K., Russell A.W., Malik R.A., Efron N. (2014). Fully automated, semiautomated, and manual morphometric analysis of corneal subbasal nerve plexus in individuals with and without diabetes. Cornea.

[23] Engelmann S., Ruewe M., Geis S., Taeger C.D., Kehrer M., Tamm E.R., Bleys R., Zeman F., Prantl L., Kehrer A. (2020). Rapid and Precise Semi-Automatic Axon Quantification in Human Peripheral Nerves. Sci. Rep..

[24] Kallinikos P., Berhanu M., O’Donnell C., Boulton A.J., Efron N., Malik R.A. (2004). Corneal nerve tortuosity in diabetic patients with neuropathy. Invest. Ophthalmol. Vis. Sci..

[25] Bandeira F., Yusoff N.Z., Yam G.H., Mehta J.S. (2019). Corneal re-innervation following refractive surgery treatments. Neural Regen Res..

[26] Petropoulos I.N., Manzoor T., Morgan P., Fadavi H., Asghar O., Alam U., Ponirakis G., Dabbah M.A., Chen X., Graham J. (2013). Repeatability of in vivo corneal confocal microscopy to quantify corneal nerve morphology. Cornea.

[27] He J., Bazan N.G., Bazan H.E.P. (2010). Mapping the entire human corneal nerve architecture. Exp. Eye Res..

[28] Turuwhenua J.T., Patel D.V., McGhee C.N. (2012). Fully automated montaging of laser scanning in vivo confocal microscopy images of the human corneal subbasal nerve plexus. Invest. Ophthalmol. Vis. Sci..

[29] Edwards K., Pritchard N., Gosschalk K., Sampson G.P., Russell A., Malik R.A., Efron N. (2012). Wide-field assessment of the human corneal subbasal nerve plexus in diabetic neuropathy using a novel mapping technique. Cornea.

